# European Hypertension Guidelines: Similarities and What the Practicing Physician Should Keep in Mind

**DOI:** 10.3390/jcm15020859

**Published:** 2026-01-21

**Authors:** Maria Elena Zeniodi, Thomas Tsaganos, Ariadni Menti, Aikaterini Komnianou, Anastasios Kollias, Emelina Stambolliu

**Affiliations:** Third Department of Medicine, School of Medicine, Hypertension Center STRIDE-7, Sotiria Hospital, National and Kapodistrian University of Athens, 11527 Athens, Greece; zeniodi@yahoo.gr (M.E.Z.); tsaganos@gmail.com (T.T.); ariamenti@yahoo.gr (A.M.); katiakomn95@gmail.com (A.K.); melina.stambolliu@gmail.com (E.S.)

**Keywords:** hypertension, ESH, ESC, guidelines, comparison

## Abstract

The European Society of Hypertension (ESH) and the European Society of Cardiology (ESC) have recently released separate guidelines for the management of arterial hypertension, published less than 12 months apart. Many practicing physicians, especially in the primary care setting, might find it challenging to thoroughly read the two lengthy documents and, most importantly, might get confused in areas of discrepancies. This review compares the two sets of recommendations using the **BEST framework**, which focuses on **B**lood pressure (BP) measurement and monitoring, **E**stablishing the diagnosis and classifying hypertension, **S**tratified patient assessment, and **T**herapeutic decisions, providing a structured overview of their areas of agreement and divergence and aiming at highlighting what the practicing physician should keep in mind. In general, the main recommendations made by the 2023 ESH and 2024 ESC guidelines regarding hypertension diagnosis and management present many similarities: office diagnostic threshold at 140/90 mmHg (multiple measurements and visits), primary role of out-of-office BP monitoring in confirming hypertension diagnosis and in follow-up of treated patients, cardiovascular (CV) risk assessment based on risk calculators and risk modifiers, initiation of drug treatment based on BP level and CV risk, treatment strategy based on steps and combination therapy, and treatment target for most patients of <130/80 mmHg.

## 1. Introduction

According to the World Health Organization, 1.28 billion individuals aged 30–79 years worldwide are hypertensive, rendering hypertension the most common cardiovascular (CV) risk factor and the most infamous target for prevention efforts [1]. In 2003, under the coordination of Professor Alberto Zanchetti, the European Society of Hypertension (ESH) and the European Society of Cardiology (ESC) jointly published the first European guidelines for the management of arterial hypertension [2]. Subsequently, the two scientific organizations collaborated in the generation of joint guidelines in 2007, 2013, and 2018.

After the two societies decided not to continue their collaboration, the ESH issued independent guidelines in 2023 [3], followed by the ESC in 2024 [4]. Both documents are intended for practicing physicians and hypertension experts and aim to support evidence-based decision-making across the continuum of hypertension care, from diagnosis to treatment.

This review attempts to present the similarities and discuss the divergences in the recommendations from the two European societies that could substantially affect physicians’ decisions and patients’ management. To present this comparison in a structured way, we introduce the **BEST FRAMEWORK**, which encompasses four key domains: **B**lood pressure (BP) measurement and monitoring, **E**stablishing the diagnosis and classifying hypertension, **S**tratified patient assessment, and **T**herapeutic decisions (Table 1).

## 2. BEST Framework for Comparison of ESH and ESC Hypertension Guidelines

### 2.1. Blood Pressure Measurement and Monitoring

#### 2.1.1. Blood Pressure Monitoring Devices

Accurate BP measurement is essential for establishing a reliable diagnosis and guiding the treatment of hypertension [5]. Both societies recommend the oscillometric method using a validated BP monitor and an appropriately sized upper-arm cuff for both office and out-of-office BP measurements [ambulatory BP monitoring (ABPM); home BP monitoring (HBPM)]. Lists of validated devices can be found in online sources (https://www.stridebp.org/ (accessed on 5 December 2025), https://www.validatebp.org (accessed on 5 December 2025)) [3,4]. A universal standardized validation protocol for BP monitors has been adopted by the US Association for the Advancement of Medical Instrumentation, the ESH, and the International Organization for Standardization (AAMI/ESH/ISO validation protocol) since the 2018 ESC/ESH joint guidelines on the management of arterial hypertension [6,7]. The ESH insists on specifically automated electronic BP monitors, while the ESC does not provide a specific recommendation.

#### 2.1.2. Blood Pressure Measurement Methods

##### Office Blood Pressure Measurement

Office BP measurement (OBP), despite its limitations, remains the reference method for hypertension diagnosis [3,4,5,7,8]. Therefore, both societies highlight the importance of the implementation of a standardized protocol in the assessment of OBP: patients should be seated for at least 5 min, in right position, and under specific conditions, and three BP readings at 1–2 min interval should be obtained [3,4]. Screening for postural hypotension is only recommended by the ESH for specific groups (people > 65 years, treated hypertensives, patients with diabetes or neurodegenerative diseases, and those with symptoms suggestive of postural hypotension), whereas the ESC recommends universal screening at the initial visit. Evaluation for systolic BP (SBP) inter-arm difference should also be performed at the initial visit, as suggested by both societies [3,4]. According to the ESH, an inter-arm SBP difference of >15–20 mmHg warrants further investigation for atheromatous or congenital arterial disease, while the respective threshold for the ESC is >10 mmHg. The arm with the highest readings should be used for subsequent measurements [3,4,9].

##### Out-of-Office Blood Pressure Measurement

It is widely recognized that ABPM and HBPM provide complementary information and have several advantages over OBP measurements [10,11,12,13]. Their value is acknowledged by both societies, which recommend their wide use, following the same measurement principles as OBP [3,4]. According to the ESC guidelines, out-of-office BP measurements have a fundamental role in all steps of the management of the hypertensive patient, i.e., diagnosis confirmation and follow up [4]. In the ESH guidelines, out-of-office BP monitoring methods are stated to have an additive prognostic value to OBP measurements and are also useful for diagnosing hypertension phenotypes, i.e., white coat, masked hypertension, and true resistant hypertension [3]. Of note, both societies recommend them for long-term management of the hypertensive individuals.

#### 2.1.3. Special Considerations

##### Blood Pressure Measurement in Atrial Fibrillation

Hypertension is the most common risk factor for the development of atrial fibrillation (AF), and this relationship is largely attributed to advanced age [14,15]. Accurate BP estimation is crucial in this population, but it could also be challenging due to increased beat-to-beat BP variability [16]. The 2018 ESC/ESH guidelines recommended multiple BP readings (no less than three) obtained by auscultation [7]. This principle is maintained in the most recent 2023 ESH and 2024 ESC guidelines, but the ESH additionally supports that the automated oscillometric method should not be excluded in AF patients, incorporating recent evidence from a meta-analysis and validation studies demonstrating the accurate estimation of SBP, which is clinically important in older people, and the moderate overestimation of diastolic BP (DBP) [16]. Since the oscillometric method is the only one indicated for out-of-office BP measurements, this represents a significant update in the ESH guidelines [3].

### 2.2. Establishing the Diagnosis and Classifying Hypertension

#### 2.2.1. Screening for Hypertension

Since hypertension is mostly an asymptomatic disease, the identification of patients with elevated BP values heavily relies on systematic or opportunistic screening. While evidence shows that screening programs improve the detection rate of hypertension, there is uncertainty about whether these programs actually improve CV outcomes [17]. Nevertheless, both societies recommend screening for hypertension in all adults, while there is variation in the recommendation about the optimal frequency of screening [3,4]. The ESC suggests annual screening for all individuals ≥ 40 years and those with elevated BP who do not meet the criteria for treatment initiation and at least every 3 years for adults < 40 years [4]. On the other hand, the ESH recommends opportunistic screening for hypertension in all adults and regular BP measurements from the age of 40 or earlier in adults at high risk for hypertension (preeclampsia, obesity, special ethnic groups, etc.) or if other CV disease (CVD) risk factors (diabetes, etc.) are present. Nevertheless, clear screening intervals are not proposed, supporting that the screening schedule should be determined by BP levels and overall CV risk [3].

#### 2.2.2. Diagnosis and Confirmation of Hypertension

The diagnosis and classification of hypertension have traditionally relied on OBP, since the largest body of evidence on the relationship between BP levels and major CV outcomes is derived from clinical trials using OBP despite its known limitations. Thus, both guidelines continue to recommend OBP as the initial and basic step for hypertension diagnosis [3,4]. The ESH recommends that abnormal OBP values should be confirmed in at least two clinic visits, unless severe hypertension [grade 3, symptoms, hypertension-mediated organ damage (HMOD), CVD] is present [3]. Moreover, physicians are encouraged to utilize out-of-office measurements to add to the prognostic value of OBP [3]. On the other hand, the ESC recommends that abnormal OBP values should be confirmed, preferably with out-of-office measurements, and if this is not feasible, repeated OBP measurements are advised [4]. This recommendation is in line with the previous 2018 ESH/ESC joint guidelines [7].

#### 2.2.3. Classification of Hypertension

In agreement with the 2018 ESH/ESC joint guidelines, it is recommended by both the ESH and ESC that the threshold for the diagnosis of hypertension should remain ≥140 mmHg and/or ≥90 mmHg for SBP and DBP, respectively [3,4,7]. This threshold is supported by robust evidence demonstrating benefits from BP-lowering treatment above this specific level [18,19,20].

The ESH maintained the previous classification of optimal, normal, and high-normal BP for values lower than 140/90 mmHg, while for complete hypertension characterization, in addition to grades (grades 1 to 3), the stages (stages 1 to 3) were incorporated based on the presence of HMOD, diabetes, CVD, or chronic kidney disease (CKD) [3].

**Figure 1:**
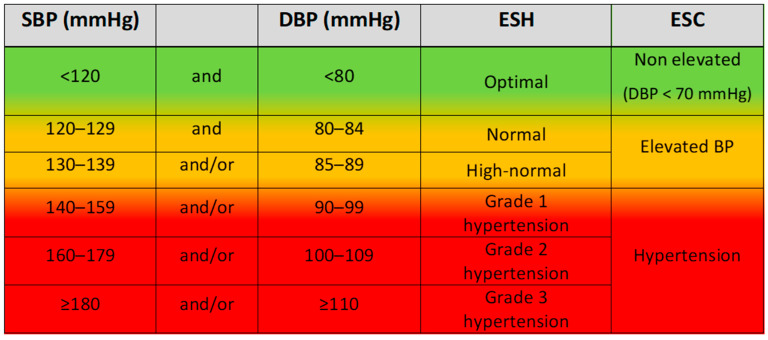
Comparison of the 2023 ESH and 2024 ESC guidelines office blood pressure classifications. SBP, systolic blood pressure; DBP, diastolic blood pressure.

On the other hand, the ESC introduced a new classification and divided BP values lower than 140/90 mmHg into two broad categories [4]: the “elevated BP” category, defined as OBP 120–139/70–89 mmHg, and the “non-elevated BP” category, defined as <120/70 mmHg (Figure 1). The rationale behind this novel classification was to identify individuals within the “elevated BP” group who have a sufficiently high CV risk to qualify them for antihypertensive drug treatment. This was based on evidence that there is a continuous association between BP values and CVD and total mortality outcomes, even in the so-called normal or high-normal range [21,22,23,24,25]. The “non-elevated BP” group mainly consists of people without elevated CV risk and only a small proportion of individuals with high risk for whom data on treatment benefits are scarce [4].

### 2.3. Stratified Patient Assessment

#### 2.3.1. Cardiovascular Risk Estimation

Within an individualized medicine framework, each patient should be stratified according to their personalized CV risk. Certain conditions are known to confer a high or very high CV risk, such as established CVD, CKD, long-standing or complicated diabetes, severe HMOD (e.g., left ventricular hypertrophy), or the presence of a markedly elevated single risk factor [3,4]. The ESC additionally includes familial hypercholesterolemia among these high-risk conditions [4]. The presence of at least one of these factors automatically classifies a person into a high or very high CV risk category that warrants a more aggressive treatment approach. In people who do not present with any of these conditions, both societies recommend estimating the 10-year risk of CVD using validated computerized risk estimation models such as SCORE2 (Systematic Coronary Risk Evaluation 2) and SCORE2 OP (Systematic Coronary Risk Evaluation 2—Older Persons) in order to guide treatment decisions [3,4,26,27]. This is particularly important in people with elevated/high-normal BP or grade 1 hypertension [3]. Hence, people with BP values within the “normal” range or slightly above the hypertension threshold but with a high CV risk would be undertreated if BP values alone were taken into account. However, the main pitfall of these risk estimation models is that they do not include several established CV risk factors. These non-traditional risk factors are either shared by both women and men or they are sex specific. The ESC has decided to include these risk factors, named as “risk modifiers”, in the estimation of the total CVD risk [4]. Female-specific risk modifiers are related to gestation and the peripartum period (e.g., gestational diabetes, gestational hypertension, and pre-eclampsia) [28,29], while shared modifiers include the following: race/ethnicity with a high CVD risk, family history of premature onset atherosclerotic CVD, socio-economic deprivation, auto-immune inflammatory conditions, etc. [4]. It is recommended that these modifiers be used to up-classify individuals with elevated BP and borderline increased predicted 10-year CV risk (5–<10%) to “sufficiently high risk”, influencing treatment decisions [4].

Finally, both societies suggest screening for HMOD, particularly in individuals at apparently low-moderate risk and/or those with an estimated 10-year CV risk < 10% or younger than 40 years, in whom the SCORE2 is not applicable, to guide treatment decisions [3,4]. The main recommended tests are serum creatinine, eGFR, and urine albumin-to-creatinine ratio to assess renal function and a 12-lead electrocardiogram to evaluate cardiac involvement. More extensive screening may include, among others, carotid artery ultrasound, carotid-femoral or brachial-ankle pulse wave velocity, and echocardiography [3,4]. The ESH also notes that speckle tracking echocardiography is a valuable tool to detect early subclinical left ventricular systolic dysfunction [3,30,31].

#### 2.3.2. Patient Selection for Drug Treatment

Both guidelines recommend lifestyle modification for all hypertensive individuals. The 2024 ESC guidelines recommend initiating pharmacological treatment at diagnosis, regardless of CV risk or age, in people with confirmed hypertension (BP ≥ 140/90 mmHg) [3,4]. Moreover, the ESC suggests a more complex approach to the selection of patients eligible for treatment rather than solely relying on BP values. Specifically, among individuals at “sufficiently high risk” with a 10-year CV risk ≥ 10% or established CVD, diabetes, CKD, familial hypercholesterolemia or HMOD, pharmacologic treatment should be initiated if BP ≥ 130/80 mmHg after 3 months of poorly implemented or ineffective lifestyle changes, while intensifying lifestyle interventions is preferred in those with BP 120–129/70–79 mmHg [4]. The same applies to people with borderline increased 10-year CV risk (5–<10%) who can become up-classified if they have coexisting sex-specific or shared non-traditional risk modifiers [4]. Exceptions are older (≥85 years) and significantly frail people [4].

The 2023 ESH guidelines also recommend initiating antihypertensive treatment in all hypertensive patients irrespective of CV risk, but there is a special note for the subgroup of patients in the lower BP range of grade 1 hypertension with no HMOD and low CV risk (i.e., no CKD, CVD, or diabetes), who might be considered for initial management with only lifestyle interventions and delay of pharmacologic treatment for up to 3–6 months [3]. Moreover, the ESH advises against treating individuals with high-normal BP and low to moderate CV risk; yet, in those at very high CV risk with established CVD, BP-lowering drug treatment should be initiated [3]. Finally, pharmacological therapy is recommended to be initiated at a higher SBP (>160 mmHg) for patients aged ≥80 years, though lower levels may be considered on an individualized basis [3].

#### 2.3.3. Evaluation for Secondary Hypertension

Although the exact prevalence of secondary hypertension is not clear, estimates range from 10% to 35%, depending on the underlying cause, and may even reach 50% in true resistant hypertension [32,33,34]. Identifying secondary causes of hypertension is crucial, as many of these conditions are potentially curable, offering the possibility of complete BP normalization [35,36,37].

A thorough investigation for clinical signs of secondary forms of hypertension has always been recommended as part of the basic work-up of a hypertensive patient [3,4,7], while, according to the ESH, further specific diagnostic evaluation should be prompted upon clinical suspicion, given that general screening for secondary hypertension is not cost effective or feasible [5]. Nonetheless, in the latest guidelines, the ESC suggests screening for primary aldosteronism—by assessing plasma renin and aldosterone levels—in all adults with confirmed hypertension [4].

### 2.4. Therapeutic Decisions

#### 2.4.1. Antihypertensive Pharmacological Treatment

Since the 2018 ESH/ESC joint guidelines, there has been a shift toward initiating antihypertensive treatment with a two-drug combination therapy in most patients, with a few exceptions [7]. This approach aims to enhance BP control by leveraging the complementary effects of different drug classes, reduce adverse effects, improve adherence, and address clinical inertia [38,39,40,41,42,43,44,45]. Moreover, in this direction, both societies acknowledge that polypills combining fixed doses of BP-lowering agents, lipid-lowering therapy and/or aspirin, if available, represent an effective strategy for CV risk reduction, as demonstrated by real-world evidence [3,4,46].

Both the 2023 ESH and 2024 ESC guidelines maintain the recommendation for initial treatment with a dual combination including two of the following first-line drug categories: a renin–angiotensin system (RAS) blocker, a calcium-channel blocker, and a thiazide/thiazide-like diuretic [3,4,47,48]. The ESH prioritize RAS blockers as an essential component of the combination, while the ESC recommends a low-dose combination of any two of these drugs, although it is stated that RAS blockers are preferred.

Although the drug choices are substantially aligned, the treatment algorithms slightly differ in sequencing. The ESH recommends starting with a low-dose combination and increasing to a full dose as a second step before adding a third drug, if needed. In contrast, the ESC recommends adding a third agent and afterwards up-titrating to full doses, if necessary. Monotherapy is recommended by both societies for frail and older people [3,4]. The ESH also recommends monotherapy in individuals with low-CV-risk BP and only modestly elevated above the BP threshold and those with very high-CV-risk and high-normal BP, whereas the ESC also recommends monotherapy in individuals with elevated BP or symptomatic orthostatic hypotension [3,4].

Both societies recommend the use of beta blockers (BBs) at any step if there is a specific indication, such as post-myocardial infarction, angina, heart failure, need for rate control (in AF), or pregnancy. However, the ESH presents BBs as first-line agents, while the ESC is against because of their inferiority in preventing stroke events.

In cases of resistant hypertension—uncontrolled BP even with a triple-drug combination, including a diuretic, at maximum tolerated doses—both societies recommend evaluation for treatment adherence, subsequent referral to specialized centers, and addition of a fourth drug [3,4]. The fourth drug should be spironolactone if eGFR ≥ 30 mL/min/1.73 m^2^ and if tolerated, although caution is warranted in patients with eGFR < 45 mL/min/1.73 m^2^ and a potassium concentration ≥ 4.5 mmol/L, since treatment-associated hyperkalemia is higher in this group of patients [3,4]. Subsequently, the ESH offers a stepwise approach, recommending the addition of vasodilating BB, alpha-1 blocker, or a centrally acting agent [3]. The ESC recommends the use of a BB as a fifth drug in case of uncontrolled BP with spironolactone or eplerenone. The addition of other drug classes is reserved for cases where further treatment intensification is required [4]. Novel treatments for resistant hypertension, including aldosterone synthase inhibitors, have emerged as promising strategies but are still under investigation [3,4,49]. Lastly, both societies endorse the consideration of renal denervation in patients with resistant hypertension and uncontrolled BP, which should be performed in specialized centers and on a shared risk–benefit discussion and multidisciplinary assessment basis [3,4].

Regarding the timing of drug administration, the ESH states that, in the general hypertensive population, morning or bedtime dosing results in a similar outcome, but antihypertensive medications should be taken in the morning for better adherence, whereas the ESC suggests administration at the most convenient time for the patient [3,4].

#### 2.4.2. Blood Pressure Treatment Targets

Evidence from recent randomized controlled trials has shown a benefit in CV risk and outcomes from a tighter BP control compared to the classic target of <140/90 mmHg in most hypertensive patients [50,51,52,53,54,55,56,57]. The ESH recommended, as a first objective for BP, a target of <140/80 mmHg in most hypertensive patients, and if drug treatment is well tolerated, the target should be <130/80 mmHg but not <120/70 mmHg in all individuals aged 18–64 years and in most up to 79 years old. This recommendation was mainly based on evidence from meta-analyses showing a reduction in CV outcomes and all-cause mortality with treatment targets for office SBP within 130–139 compared to ≥140 mmHg and a further increase in benefit with SBP values of 120–129 and DBP values <80 mmHg compared to 80–90 mmHg and from data showing higher treatment discontinuation rates with lower BP values [19,58,59]. In patients over 80 years old, it is recommended that SBP values within the 140–150 mmHg range are desirable, though SBP of 130–139 mmHg could be considered if treatment is well tolerated. The ESC adopted a general BP treatment target of 120–129/70–79 mmHg, if tolerated [4]. Of note, taking into account that research data are not always applicable in routine clinical practice and BP-lowering treatment is not always well tolerated, the ESC recommend applying the “ALARA principle”: “as low as reasonably achievable” [4]. Regarding people older than 85 years or those who are significantly frail, there is inconclusive evidence; hence, a more personalized BP-lowering approach is recommended without further special mention by the ESC [4].

## 3. Conclusions

The primary goal of arterial hypertension guidelines is to support clinicians in improving patient outcomes through effective, evidence-based management. Despite the availability of multiple guidelines documents over two decades, hypertension control rates remain suboptimal, reflecting gaps in implementation, adherence, and clinical inertia [60,61,62].

The 2023 ESH and 2024 ESC guidelines share substantial overlap in diagnostic methods, risk stratification, and treatment principles. However, some differences exist, most notably the ESC’s more aggressive BP initiation treatment thresholds and less lenient BP control target, especially in older people, its broader use of risk modifiers and out-of-office measurements, as well as the new BP classification. With this new classification, many more patients with BP levels in the range of 130–139/80–89 are eligible for drug treatment if the CV risk is high or very high, whereas, according to the ESH, this recommendation regards only those with established CVD. Moreover, it may facilitate earlier identification of CV risk and promote timely lifestyle interventions, particularly in younger individuals and those at intermediate risk. Clinicians should take into account that standardized office and out-of-office BP measurements, along with a careful assessment of the CV risk, constitute the basis for the optimal management of hypertension. Patient-centered care remains the cornerstone of hypertension management, emphasizing individualized treatment decisions based on overall CV risk, comorbidities, and patient preferences. In this context, as also highlighted by the ESH, there is a potential future role of artificial intelligence, as advanced algorithms may support clinicians in optimizing risk stratification, tailoring therapy, and improving adherence, thereby enhancing the effectiveness of personalized hypertension management.

## Figures and Tables

**Table 1:** Comparison of the 2023 ESH and 2024 ESC Guidelines (**BEST**: **B**lood pressure measurement and monitoring, **E**stablishing the diagnosis and classifying hypertension, **S**tratified patient assessment, and **T**herapeutic decisions).

	Area of Comparison	2023 ESH Guidelines	2024 ESC Guidelines
**B**	BP monitors	Validated upper-arm cuff BP device; appropriate cuff	
	OBP methodology	3 readings per visit; 5 min rest	
	Inter-arm office SBP difference requiring evaluation	>15–20 mmHg	>10 mmHg
	Out-of-office BP measurements	Strongly encouraged	Recommended
	BP measurement in AF	Oscillometric/Auscultation	Auscultation
**E**	Screening	• Regular BP measurements: >40 years, <40 years if high CV risk • Screening interval not set	• Annually: >40 years, untreated with elevated BP • Every 3 years, at least: <40 years
	Diagnosis/Confirmation	• OBP at least 2 visits (4 weeks apart) unless severe hypertension • ABP/HBP additive prognostic value	• OBP + ABP and/or HBP • Repeated OBP if ABP/HBP unfeasible
	Classification	• Optimal • Normal • High normal • Hypertension (grades 1, 2, and 3 plus stages 1, 2, and 3)	• Non-elevated • Elevated • Hypertension
**S**	CV risk estimation	• High risk conditions • SCORE2, SCORE2-OP • Risk modifiers	
	Patient selection for drug treatment	• ≥130/80 mmHg + CAD • ≥140/90 mmHg • ≥160 mmHg if ≥80 years	• ≥130/80 mmHg + CVD, DM, FH, CKD, HMOD, 10-year CV risk ≥ 10% • ≥130/80 mmHg + 10-year CV risk 5–<10% combined with risk up-classification according to non-traditional risk modifiers • ≥140/90 mmHg
	Secondary hypertension	• Disease-guided diagnostic evaluation upon clinical suspicion	• Screening for primary aldosteronism in all hypertensives
**T**	Pharmacological treatment steps	1. Low-dose dual combination with RAS blocker + CCB or TZD/TZD-like 2. Increase to full dose 3. RAS blocker + CCB + TZD/TZD-like	1. Low-dose dual combination with any two drugs from RAS blocker/CCB/TZD/TZD-like 2. RAS blocker + CCB + TZD/TZD-like 3. Increase to full dose
	Pharmacological treatment monotherapy	1. ≤150/95 mmHg + Low CV risk 2. 130–139/80–89 mmHg + High CV risk 3. Frail and/or old	1. 130–139/80–89 mmHg 2. Symptomatic orthostatic hypotension 3. Frail and/or old
	On-treatment BP target	1. <130/80 mmHg in most hypertensives up to 79 years old 2. 140–150 mmHg if ≥80 years old	120–129/70–79 mmHg

OBP, office blood pressure; BP, blood pressure; AF, atrial fibrillation; CV, cardiovascular; ABP, ambulatory blood pressure; HBP, home blood pressure; RAS, renin–angiotensin system; CCB, calcium-channel blocker; TZD, thiazide; CAD, coronary artery disease; HMOD, hypertension-mediated organ damage; DM, diabetes mellitus; CKD, chronic kidney disease; FH, familial hypercholesterolemia; SCORE2, Systematic Coronary Risk Evaluation 2; SCORE2-OP, and Systematic Coronary Risk Evaluation 2—Older Person.

## Data Availability

No new data were created or analyzed in this study.

## Abbreviations

OBP	office blood pressure
BP	blood pressure
AF	atrial fibrillation
CV	cardiovascular
ABPM	ambulatory blood pressure measurement
HBPM	home blood pressure measurement
RAS	renin-angiotensin system
CCB	calcium-channel blocker
TZD	thiazide
HMOD	hypertension mediated organ damage
CKD	chronic kidney disease
SCORE2	Systematic Coronary Risk Evaluation 2
SCORE2-OP	Systematic Coronary Risk Evaluation 2–Older Persons
